# A Methodology Towards Mechanical Properties Optimization of Three-Component Polymers by the Gradual Variation of Feed Composition in Semi-Continuous Emulsion-Free Radical Polymerization

**DOI:** 10.3390/polym11122125

**Published:** 2019-12-17

**Authors:** Francisco J. Rivera-Gálvez, Luis J. González-Ortiz, Miguel A. López-Manchado, María E. Hernández-Hernández, Carlos F. Jasso-Gastinel

**Affiliations:** 1 Chemical Engineering Department, Universidad de Guadalajara, Blvd. Gral. Marcelino García Barragán, 1421, Guadalajara 44430, Jalisco, Mexico; franciscodr10@outlook.com (F.J.R.-G.); maelena.hernandez@gmail.com (M.E.H.-H.); 2 Chemistry Department, Universidad de Guadalajara, Blvd. Gral. Marcelino García Barragán, 1421, Guadalajara 44430, Jalisco, Mexico; 3 Institute of Polymer Science and Technology, ICTP-CSIC, Juan de la Cierva, 3, 28006 Madrid, Spain; lmanchado@ictp.csic.es

**Keywords:** semi-continuous, methodology, three-component, gradient composition, multicomponent polymers

## Abstract

In this work, a new methodology for the synthesis of three-component polymers (TCPs) was developed using a seeded, semi-continuous free-radical emulsion polymerization towards the optimization of the moduli–ultimate deformation performance and energy dissipation capacity for a styrene (S), n-butyl acrylate (BA), and 4-vinylbenzyl chloride (VBC) system. The three components were sequentially fed in pairs, varying feed composition along the conversion using S as the common monomer. To prepare a reference material, an industrial method was utilized with those monomers, using an equivalent global composition in a two-stage batch process (TS). Nanophase formation in the particles was observed by transmission electron microscopy (TEM), while the separation of the phases in the solid samples was observed by atomic force microscopy (AFM). The changes in glass transition temperature were determined by differential scanning calorimetry (DSC) and dynamic mechanical analysis (DMA). The latter was primarily used to compare mechanodynamic properties as a function of temperature for the two synthesis methods used. Thus, the higher toughness of the forced composition three-component polymeric materials was evaluated by means of their energy dissipation capacity, toughness, and stress–strain measurements at several temperatures.

## 1. Introduction

In the search for synergistic interactions among the components of polymeric materials to optimize their mechanical properties, gradients in composition have been sought for different types of polymeric materials [1,2,3]. For two-component polymers, the possibilities are very vast, since in blends and copolymers, the chemical systems and, therefore, their properties, can be widely modified. Looking for gradients in mechanical properties, the idea succeeded in blends since some time ago [4]; in slightly crosslinked blends (interpenetrating polymer networks), it was demonstrated that attaining a spatial gradient in composition and limiting phase separation at the microscopic level, the maximization of the contribution of each high molecular weight polymeric component can be achieved [4]. That is, the properties contribution of each component is optimized; if the gradient composition is achieved, some region(s) are rich in component “A”, and other region(s) are rich in component “B”, provided that a close interaction is being maintained among those components by means of a gradual change in composition within the polymer bulk. The high molecular weight allows a full contribution of the specific characteristics of a polymeric component [1,4]. Such gradual change allows the presence of regions with intermediate composition which promotes the interaction of the components and diminishes phase separation [5].

The key to extending those component contribution principles to copolymers obtained by free-radical polymerization (FRP) is to vary, in gradual form, the composition of high molecular weight copolymer chains that are formed instantaneously along conversion (which is not the case for polymers obtained by reversible-deactivation radical polymerization (RDRP), where the chains grow as conversion progresses). In that way, a certain distribution of the chains’ composition can be formed, where the chains rich in the repeating unit (RU) “A” can be formed first, and, later in the reaction, the chains rich in the RU “B” can also be synthesized. The desired changes in the composition of the copolymer chains to be formed can be reached in a semi-continuous reactor, considering feeding profiles, provided that the monomers involved in the reaction do not show the tendency to form homopolymers (i.e., statistical copolymers can be formed).

The gradual change in composition allows the interaction of the components and contributes to avoiding a substantial phase separation as it happens, for example, in core–shell-type polymers (which are synthetized in two stages).

Such gradual change in chains’ composition using a semi-continuous reaction can be readily performed in solution or emulsion; however, the emulsion process is more convenient, because it is preferable to use water rather than an organic solvent to control the heat that is released in the reaction by the double bond breakage of the comonomers as the conversion proceeds.

In fact, the aforementioned referred composition variation has already been demonstrated, using the styrene/n-butyl acrylate system (70/30 wt %), where a considerably higher damping capacity was obtained with this type of copolymeric materials, compared to a traditional core–shell type polymer [6]; furthermore, for the same chemical system, using a 50/50 wt % global composition, much higher Young’s modulus and toughness values were obtained with these forced composition copolymers (FCC), compared to equivalent statistical and two-stage polymeric materials [7]. The evolution of chains’ composition along conversion (for the FCC) was followed by proton nuclear magnetic resonance (^1^H NMR). In those works, the structure–properties relationship was clearly disclosed, “building histograms” of the distribution of copolymer chain’s composition contained in each polymer system [6,7,8]. In fact, the great potential to design the properties of a copolymer using that method was demonstrated, disclosing the different types of stress–strain behavior that can be obtained by variations in the feeding profiles while maintaining constant the global copolymer composition [7]. Briefly, the high modulus along with significant values in ultimate deformation that can be obtained denote that a synergistic performance can be achieved with that methodology [8,9].

For the mechanical performance rationalization of this type of copolymer system [7], the principles explained previously also apply to copolymers synthesized by RDRP, where a gradient in composition is formed in each chain [10,11], and optimal synthesis procedures for linear gradient copolymers have been proposed [12]. Reviews with that technique have shown subsequent advances in that field [13,14,15].

The relevance of the aforementioned principles for the properties contribution of the components that apply to the two copolymer synthesis methods just mentioned (i.e., FRP and RDRP), has been demonstrated by means of the similar results that have been obtained with both methods, evaluating dynamic properties for styrene-butyl acrylate copolymers [8,10] as well as for stress–strain behavior [16].

As an extension, looking for methods to improve properties in three-component polymers (TCPs), the strategy to force a variation in chains’ composition along the reaction using FRP can also be applied to polymer systems with three components, where the instantaneous composition of the polymer chains can be adjusted by feeding co-monomeric pairs (e.g., feeding A and B monomers first and then monomers A and C for a three-component system) to avoid the well-known relative reactivity problems that arise if three or more monomers are fed simultaneously. The chains’ composition along each copolymerization can be oriented, taking as a guide previous reports [7,8], using variations in feed composition of each comonomer system to be fed.

Based on the former reasonings relative to the effects obtained by forcing the copolymers’ composition [1,6,7,8,9], in this work, a methodology for the synthesis of a three-component polymer material is proposed, using a seeded, semi-continuous free-radical emulsion polymerization aimed at changing the moduli and deformation capacity to improve toughness (i.e., mechanical properties optimization) with respect to the material formed by an industrial method [17], using the styrene (S), n-butyl acrylate (BA), and 4-vinylbenzyl chloride (VBC) system, where the three components are sequentially fed by pairs, using S as the common monomer.

To illustrate the functionality of the method, cases of reactions’ alternating feed profile or seed type are presented. The VBC monomer was chosen as an adequate component for possible post-reactions with the chloromethyl group and its affinity with S to make a copolymer ($r_{S}=$ 0.72 and $r_{VBC}=$ 1.31 [18] while $r_{S}=$ 0.72 and $r_{BA}=$ 0.27 [19]). Due to the specific interaction of their respective chemical groups, the styrene and n-butyl acrylate system holds a low interaction parameter [20] and is considered a weakly segregating monomer pair [21] which allows the coexistence of a broad range of copolymer compositions with low tendency to phase separation. As a reference (to confront mechanical properties), a core–shell-type polymeric material was also prepared, following a typical industrial method using two stages (TS); equivalent global compositions were considered in all the synthesized polymeric materials [17]. Thus, the main expected differences among the polymers obtained with the method presented here (forced composition TCPs) and the industrial method considered as a reference (TS) were the chain composition profile being formed throughout the reaction and, as a consequence of it, the different segregation levels of the final system (e.g., different size of the formed domains).

To establish such differences, in this work, the phase morphology was disclosed depicting the nanophase separation in the polymeric materials by atomic force microscopy (AFM). The changes in glass transition temperature ($T_{g}$) were determined by differential scanning calorimetry (DSC) and were compared with values measured using dynamic mechanical analysis (DMA). The latter technique was primarily used to evaluate the mechanodynamic properties as a function of temperature. The expected higher toughness of the forced composition TCP was evaluated by means of their energy dissipation capacity, toughness, and stress–strain measurements at several temperatures.

## 2. Experimental

### 2.1. Materials

The three monomers (purchased from Sigma–Aldrich, (S–A), St. Lousi, MO, USA; purity ≥99% for S and BA and ≥90% for VBC) were disinhibited with tertbutyl catechol acquired from S–A (S and VBC)) or methyl ester hydroquinone acquired from S–A (BA). Sodium dodecyl sulfate (SDS, S–A; purity ≥99%) was used as surfactant, potassium persulfate (KPS, S–A; purity ≥99%) as initiator and sodium bicarbonate (SB, Arm and Hammer) as buffer. Distilled water (Selectropura, Guadalajara, Mexico) to prepare the emulsions and nitrogen gas (Infra; purity >98%) to promote an inert atmosphere were used in every reaction.

### 2.2. Synthesis Methodology

#### 2.2.1. Synthesis of Polymer Seeds (Polystyrene (PS) or Poly (n-Butyl Acrylate) (PBA))

A stirred batch emulsion process was carried out containing 2000 g of water, 500 g of the correspondent monomer (S or BA), 10.0 g of KPS, 10.0 g of SDS, and 10.0 g of SB. The reaction temperature was 70 ± 2 °C; the stirring rate was 400 ± 5 rpm (at the beginning of the heating procedure and during the entire reaction time); the nitrogen gas was bubbled at a constant flow for 1 h before the reaction was initiated (with initiator addition), sustaining the flow during the whole reaction (4 h).

#### 2.2.2. Synthesis of Forced Composition Three-Component Polymers

**Figure 1:**
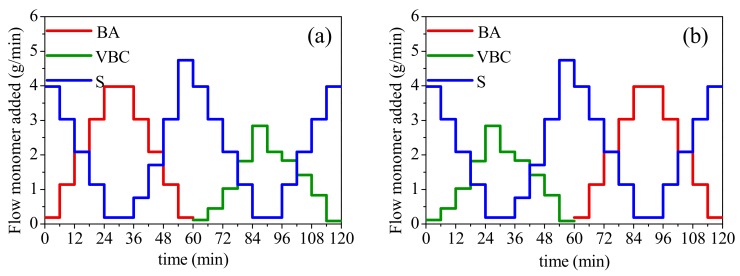
Profiles of BA, S, and VBC monomers used to prepare the three-component polymers (TCPs): (**a**) profile I; (**b**) profile II. For materials code, see the previous paragraph.

The forced composition TCPs were obtained by means of a semi-continuous seeded emulsion polymerization using some mass of the seed latex previously synthesized (PS or PBA), following the feeding schedule that is described below, maintaining the reaction system permanently stirred (400 ± 5 rpm) and thermostated (70 ± 2 °C) using a flow of nitrogen gas (initiated 1 h before the reaction was started and maintained during the entire reaction time). When the process started, 250 g of the latex (which contained 50.0 g of PS or PBA, representing 10 wt % of the total polymer mass to be obtained) along with 1400 g of distilled water was added to the reactor. To start the first stage of the reaction, 20.0 mL of a solution containing SDS, KPS, and SB was added to the reactor (the solution contained the mass of each substance that corresponded to 2 wt % of the total mass of monomers to be added in each stage); this process was repeated at the start of each of the 20 feeding stages. For each stage, the monomers were fed in pairs, following the profiles shown in Figure 1a (profile I) or Figure 1b (profile II); in accordance with previous studies for the two component systems [6,7], the total feeding time was 2 h. The comonomer feeding profiles were planned in ascendant or descendant paths of straight or parabolic trajectories. To follow conversion gravimetrically, samples were taken along the conversion. With this method, the exotherm due to the monomer conversion slowed down due to the semi-continuous process used; besides, the water of an emulsion system allows for the dissipation of heat, avoiding auto-acceleration of the reaction. After a reaction was stopped, the system was cooled to room temperature to collect the polymeric material.

All the forced composition TCPs globally contained 25/60/15 wt % of BA/S/VBC, respectively. From now on, to identify the TCP, the used profile (I or II) will be written first (Figure 1), and the seed polymer used (PS or PBA) subsequently; thus, their material codes are: IPS, IPBA, IIPS, and IIPBA.

#### 2.2.3. Synthesis of TS Polymer

For the three-component polymeric material that acted as a reference, a TS polymer containing an equivalent overall composition to that of the TCP was synthesized in two stages. For the first stage, a PBA seed latex was synthesized, as previously indicated. For the second stage, 625 g of the PBA seed latex; S and VBC comonomers were added to match the desired concentrations (125 g of PBA, 300 g of S, and 75 g of VBC). Afterward, 300 mL of an aqueous solution containing 7.5 g of SDS, 7.5 g of KPS, and 7.5 g of SB were also charged to the reactor (the amount of SDS, SB, and KPS added represented a 2 wt % of the total amount of monomer to be polymerized in the second stage) to obtain, at the end of the reaction, the TS three-component polymeric material.

The recipes for the synthesis of the different polymeric materials are shown in Table 1. The solid content of polymer product at the end of a reaction was 20 wt %.

### 2.3. Materials Processing

The synthesized latexes were dried in a chamber at environmental conditions, using a current of dry air. Every solid material was washed 3–4 times with distilled water and dried again in the chamber. Then, by manual cryogenic grinding (using liquid nitrogen) with a mortar, the respective polymeric powder was obtained. Each powder was then processed by compression molding (Carver Press model 3895) at 140–160 °C and 16 MPa in a 15 min cycle, using appropriate mold cavities to prepare samples in accordance to the required dimensions to apply the ASTM D 4065–01 or ASTM D 638–03 test for mechanodynamic and tensile tests, respectively.

### 2.4. Characterization

Thermograms were attained by means of differential scanning calorimetry (DSC, Discovery TA Instruments, New Castle, DE, USA), with 3–5 mg of solid polymer product, using 20 °C/min as heating rate in a nitrogen atmosphere.

Micrographs of processed samples were obtained by atomic force microscopy (AFM, Bruker MultiMode^®^ 8, tapping mode) after transversal cryofracturing (microtoming at −35 °C) and polishing were accomplished.

Mechanodynamic tests as a function of temperature were performed in a dynamomechanical analyzer (DMA, TA instruments Q800) using a three-point bending clamp and amplitude of 10 μm at 1 Hz (heating rate: 1.5 °C/min).

Tensile tests were carried out at 23, 40, and 50 ± 2 °C using a temperature chamber and crosshead speed of 5 mm/min with specimen type V (Instron 3366, Norwood, MA, USA).

## 3. Results and Discussion

For the forced composition TCPs, high conversions (95–98 wt %) measured by gravimetry were, obtained after 135 min of reaction (only 15 min after the feeding time ended), stopping the reactions 60 min afterwards, finding that no further conversions were obtained. Such data show reasonable reaction times for high conversion. For the TS polymeric material, a similar conversion for the second stage was achieved in 35 min.

**Figure 2:**
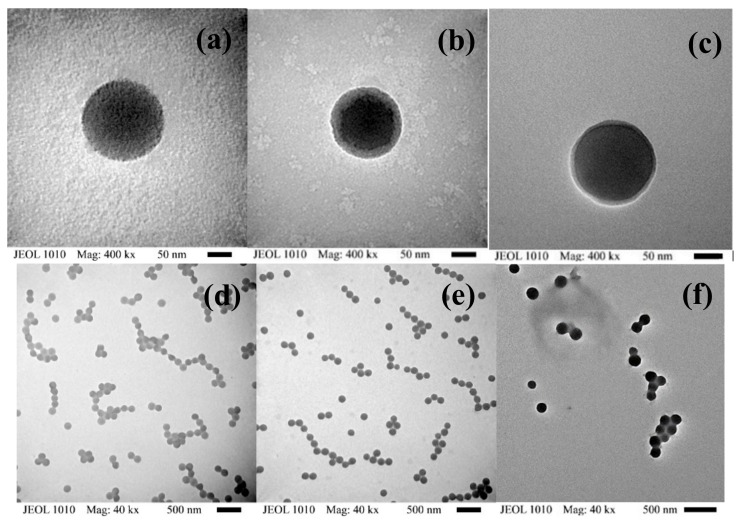
TEM images of polymer particles for TS, IPS, and IIPS at 400 kx (**a**–**c**), respectively, and similarly (**d**–**f**) at 40 kx.

Examples of synthesized polymer particle samples of the two types of polymeric materials can be observed in Figure 2a–f where a two-phase morphology can be clearly noticed in all polymer systems. In the case of the TS material (Figure 2a), the phase separation basically occurs by the incompatibility of the seed polymer (PBA) and the copolymer prepared in the second stage (P(S-co-VBC)) to make the multicomponent polymer. For the IPS and IIPS materials (Figure 2b,c), the phase separation was mainly due to the nanophase segregation that arises from the polymer chains that are formed by the sequential two pairs of comonomers, even though they share one common component. Additionally, for the TS material, a higher degree of phase separation compared to the TCP can be noticed, demonstrating that such degree can be decreased by means of the synthesis methodology proposed here.

**Figure 3:**
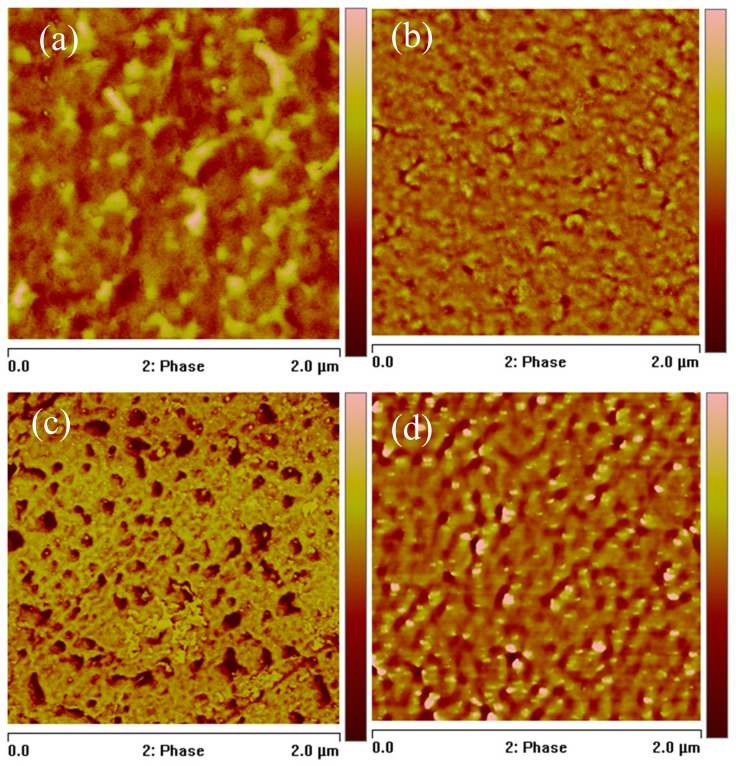
AFM images of (**a**) TS, (**b**) IPS, (**c**) IIPS, and (**d**) IIPBA.

In Figure 3, the two phases formed can be clearly noticed in all samples. In Figure 3a, two phases were formed due to the core–shell type synthesis process used for the TS material, while in Figure 3b,d, due to the seeded 20 stage sequential synthesis process utilized for the TCP, smaller nanophase domains (with respect to the reference material) were formed. This can be expected due to the gradual change in composition along conversion for IPS and IIPS. In the TCP, it is important to notice that more continuity on the phases can be observed in IPS and IIPS than in IIPBA; that may be due to the presence of the PBA seed which induces the formation of small islands that promote a decrease in phase continuity. The AFM results are in accordance with TEM observations in terms of phase separation level. Further details on phase separation and its effects on properties are discussed in the analysis of the mechanical properties performance.

The smaller domain morphology leads to a better component interaction response when a load is applied to the TCP (i.e., the contribution to modulus (favored by S and VBC repeat units) and deformation (promoted by BA units) occur in a closer framework for the interaction contribution). Such a type of morphology may lead to a synergistic response in mechanical performance (see ahead the discussion about toughness and energy dissipation capacity), because it leads to the optimization of the contribution of the components, provided that no miscibility occurs [22]. Such mechanical improvement has been demonstrated with copolymers containing a gradient in chains’ composition versus equivalent core–shell-type materials [8] or diblock copolymers [16]. Núñez et al. [8] obtained an improved static and dynamic mechanical performance (moduli and toughness) for variable composition copolymers conceptually similar to those synthesized in this work compared with equivalent two-stage polymers, even varying the seed diameter size. Guo et al. [16], using RDRP, also obtained a smaller domain size for gradient versus diblock copolymers and attained higher deformation capacity with the gradient copolymers.

**Figure 4:**
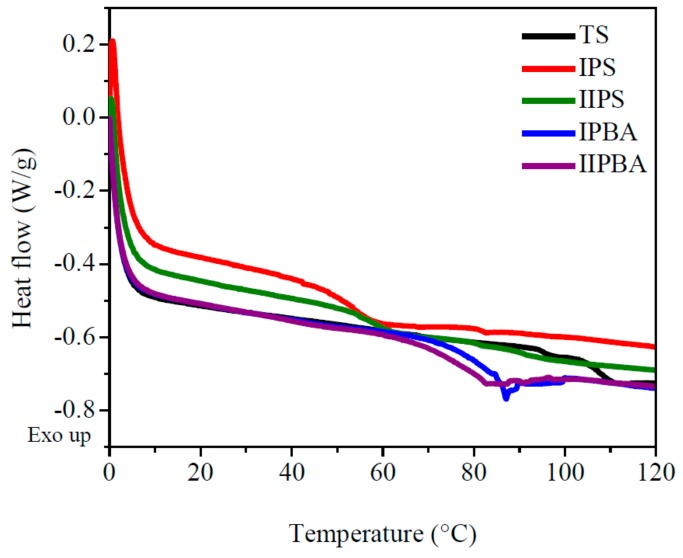
Differential scanning calorimetry analysis for TS polymer and TCPs in nitrogen atmosphere and heating rate of 20 °C/min. For materials code see Section 2.2.2.

**Figure 5:**
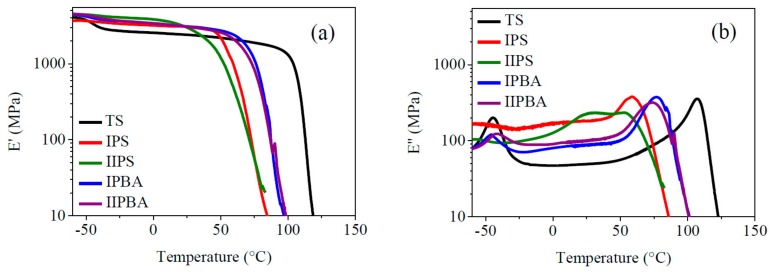
(**a**) Storage and (**b**) loss moduli versus temperature for TS polymer and TCP. Frequency of 1 Hz. Heating rate of 1.5 °C/min. For materials code see Section 2.2.2.

A DSC thermogram for the 0–120 °C interval (which covers the range for ambient and typical application temperatures) can be seen in Figure 4, where the correspondent $T_{g}$ value of the evaluated materials was detected using the midpoint method. In Table 2, those values are presented along with the values obtained from the peaks of the loss modulus plot (values for the −60 to 120 °C temperature range obtained from Figure 5b. Since $T_{g}$ is related to the segmental movement in chains (of amorphous polymer zones) which occurs as energy is applied to a sample, commonly the $T_{g}$ values of a material are reported using DSC measurements. In that way, looking at Table 2, the $T_{g}$ values determined by DSC of the different forced composition TCPs are shown in the second column. Nevertheless, for polymers containing two or more components, the temperature transitions vary significantly in accordance to the chains’ composition, their architecture, etc. For those cases, a DMA analysis is more appropriate, because it can follow precisely the whole curve of the loss modulus (E″) of the polymeric materials as a function of temperature. E″ represents the energy dissipated or lost as heat, and, thus, the $T_{g}$ of a material appears as a peak in the plot, and for copolymers or terpolymers, multiple peaks may appear (as it happens in the materials presented here). Those peaks broaden if a wide distribution of chains’ composition occurs (e.g., for copolymers where a variable composition is promoted along conversion, as is the case here). Such broad transitions and high E″ values are most effective for absorbing materials (impact or sound). Regardless of such variations in glass transition behavior, for the specific E″ peak values that are also shown in the columns 3 and 4 of Table 2, the closeness between the DSC $T_{g}$ values and the correspondent ones obtained with E″ is noteworthy. There, for the TS material, the 108 °C value corresponds to the S/VBC statistical copolymer formed in the second stage of its synthesis reaction. For the forced composition TCP, the $T_{g}$s vary with the type of seed and the feeding sequence (i.e., the type of feeding profile).

According to the seed used in the TCP, the materials with PBA seed show a higher value of the second $T_{g}$, because the global content of “BA” units ($T_{g}$ of PBA is −54 °C [23]) in the S/BA copolymer is comparatively lower (compared with the materials prepared with PS seed). Regarding the profile type used (i.e., I or II), the materials prepared with profile II showed slightly lower values of $T_{g}$, denoting that the feeding profile used promoted the synthesis of chains with a comparatively higher “BA” content in the copolymer formed in the second part of the reaction. It can also be stated that the seed type had more influence on the $T_{g}$, as it can be seen in Table 2 and Figure 5b. Further details on E″ behavior are explained in the loss modulus discussion. The presence of two widely separated glass transitions in Table 2 had to do, first, with the difference in $T_{g}$s of the PBA, PS, and PVBC (typically −54, 100, and 101 °C respectively [7,23,24]). Such differences are due mainly to the substituents pending on the vinyl group; the butyl group imparts free volume and, thus, flexibility to the chains, whereas the phenyl and chlorinated phenyl groups of the other two monomers impart rigidity to the polymer chains and are related to the second $T_{g}$ value in Table 2. Since the structure of the monomers to form the TS and TCP materials is equal as well as the polymer processing to prepare the test samples, the only differences between the TS material and the forced composition TCPs arise from the seed type and synthesis method used. These factors lead to differences in the types of chains formed throughout the respective reactions, basically P(S-co-BA) and P(S-co-VBC) for the TCPs and PBA/P(S-co-VBC) for the TS (as it can be seen in Section 2.2.2 and Section 2.2.3, respectively). For the latter, “S” repeat units promote interactions among the two types of copolymer chains; additionally, for the TCPs, the semi-continuous polymerization reaction allows the variation in the composition of the polymer chains formed along conversion which, in turn, leads to variations in the $T_{g}$ values (especially in the second $T_{g}$ of Table 2, due to the “BA” content integrated to the respective copolymer chains in the mass bulk).

The mechanodynamic behavior of all polymeric materials discussed ahead depict their behavior when very low cyclic deformation is applied, reflecting with accuracy the storage and dissipation capacity of the tested materials when energy is applied while the temperature is increased. In Figure 5a, for the storage modulus ($E^{\prime }$), it can be noticed that the TS polymer presented the highest temperature resistance before the final $E^{\prime }$ decay due to the influence of the S/VBC copolymer formed in the second stage; however, it can also be seen that it was the only material that showed a noticeable decrease in $E^{\prime }$ at low temperature (approximately −50 °C), denoting the disadvantage of having full-phase separation of its PBA component (25 wt % globally). The TS material showed the lowest $E^{\prime }$ value (compared with the TCP) along the whole temperature range (from −35 °C), until each one of the TCPs presented its own decay in the plastic zone (between 35 and 65 °C); for example, at 25 °C, the TS polymer showed an E’ value which was approximately 30% lower than the ones corresponding to the TCPs. For the TCPs, the higher the amount of S/VBC in the correspondent copolymer formed, the higher the temperature resistance before the $E^{\prime }$ decay.

The storage modulus decay shown in Figure 5a, was reflected as a peak for the loss modulus as a function of temperature (Figure 5b), that stands for the $T_{g}$ of the different polymeric materials, disclosing phase separation if multiple peaks arise. For the reference material, the PBA peaked at low temperature (around −45 °C) and the S/VBC copolymer peak (approximately 105 °C) was clearly indicated, showing the lowest E″ value among the peaks, denoting the lowest energy dissipation capacity in that region among all the synthesized polymeric materials.

For the forced composition TCPs in the low temperature region, the materials prepared with PS seed did not show a clear peak in the PBA $T_{g}$ vicinity, while the PBA seed presence was reflected with a small peak at approximately −45 °C for the other two TCPs. Nevertheless, such a peak was not combined with a noticeable decrement in $E^{\prime }$ at that temperature (Figure 5a). The peaks above room temperature denoted the useful temperature limit of those TCPs. The very broad peak of the IIPS material denotes a wide composition spectrum of the polymeric chains. Besides, comparing with the others, it showed the highest energy dissipation capacity around room temperature (e.g., 15–45 °C). However, considering the interval −60 to 50 °C, the IPS material showed the highest overall energy dissipation capacity (area under the curve) among all synthesized materials; for the specific values of energy dissipation capacity (see Table 3 after mechano-static discussion).

**Figure 6:**
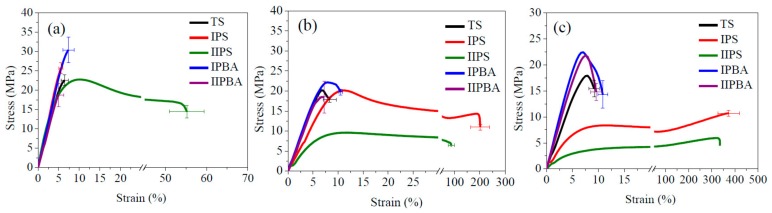
Strain–stress curves for TS polymer and TCPs at a crosshead speed of 5 mm/min and (**a**) 23, (**b**) 40, and (**c**) 50 ± 2 °C. For materials code see Section 2.2.2.

The stress–strain behavior shown in Figure 6 for those materials, allows the observation of the elastic modulus, as well as their strength and deformation capacity up to rupture at the test temperature.

In Figure 6a, the TS material at 23 °C showed a behavior similar to some of the TCP but lower rupture strength (mainly associated to higher phase separation). At that temperature, the IIPS material was the only one that showed yielding with considerable deformation capacity (approximately 55%), along with a modulus that was comparable to that of the TS polymer. That performance was in accordance with the loss modulus behavior (Figure 5b) which showed a wide peak that started at approximately 0 °C. Such a combination of high modulus and deformation stands as a target for engineering applications. Nevertheless, in this particular case, the drawback of this material is its low $T_{g}$. The absence of yielding on the other TCPs is a consequence of their relatively high $T_{g}$. In the IPS material, an even wider peak can be appreciated, between −25 and 45 °C (see Figure 5b).

At 40 °C, the Young’s modulus of the reference material remains with a high value (Figure 6b), showing a yield point with a very low deformation capacity (approximately 10%). At this temperature, the IIPS material showed an important decrement in its modulus and in its ultimate stress (in both properties, ~50% with respect to its value at 23 °C), but its deformation capacity was sustained. At 40 °C, the best combination of properties corresponded to the IPS material, because its modulus was only 14% lower than that of the reference material, but its deformation capability increased to approximately 200%. The forced composition TCPs prepared with PBA seed followed a similar trajectory to the one shown by the reference material, denoting the disadvantage of having a high content of copolymer chains with a comparatively lower BA content (as compared to those of IPS and IIPS materials).

The changes in those mechanical parameters when the temperature increased to 50 °C were not significant, neither for the reference material nor for the TCP prepared with PBA seed. Due to their high S-VBC copolymer content, the modulus value was still high and its deformation capacity was low, showing lack of interaction with the rubbery component (PBA). However, the IPBA and IIPBA materials showed higher modulus and yielding stress than the reference material which can be attributed to their smaller phase separation degree. On the contrary, the two TCPs that presented high deformation at 40 °C increased even more their deformation capacity at 50 °C (>350%) which represents an increase in toughness, especially for the IPS material which had a modulus that did not decrease as much as the IIPS material.

Measuring the area under the E″ curves (Figure 5b), the values for energy dissipation capacity between −60 and 50 °C for the different polymeric materials are shown in Table 3. It can be seen that the TS material had the lowest value (in agreement with the observed highest phase separation). For the TCP, the materials prepared with PS seed show substantial increments with respect to the TS material (241% for IPS and 208% for IIPS). For these materials, the variations in the respective trajectories of the E” value as the temperature increased (e.g., the wide peak in IIPS) were due to the differences among the respective variations in the composition of the copolymer chains formed throughout such reactions. The TCPs prepared with PBA seed show a comparatively smaller peak at their respective $T_{g}$ (compared to that one of the TS material). The last was a consequence of their lower relative “BA” contribution for phase separation with the copolymer chains rich in BA, which promoted a lower phase separation level. The lower second value of the TCP compared to the TS polymer can be attributed to a lower nanophase separation, as it is shown in Figure 3b–d and Table 2.

For toughness (measuring the area under the stress–strain curves) of the polymeric materials, the values in Table 3 are presented at 25, 40, and 50 °C. At 25 °C, since the elastic moduli are very similar, the difference in values was due to the rupture stress (higher for IPBA and deformation capacity (much higher for IIPS)). Higher differences are shown at 40 and 50 °C, where the deformation capacity of IPS and IIPS highly exceeded the other values at both temperatures. The very high values of IPS had to do with modulus, yielding stress maintenance, and high deformation (Figure 6b–d). The TS material and the TCP prepared with PBA seed did not show a significant increment in toughness, even at 50 °C. Those materials could be useful for applications, where temperature resistance is important, provided that high toughness is not required.

Looking at the mechanical performance of the TCP compared to the TS reference material (moduli and deformation capacity, along with energy dissipation and toughness), it can be stated that the novel synthesis method proposed here to prepare TCP [25] allowed variations in the relative composition among the different monomer units that form the polymeric chains and can force oriented modifications on the material to vary rigidness/elasticity, looking for specific properties [6,7,8]. Such variations in composition allow, in turn, the design of polymer properties for engineering or specific applications in accordance with the required conditions. Those achievements can be reached by designing the copolymer composition variation and selecting the seed to be used to optimize the components’ interactions while keeping phase separation at a nanoscopic level.

With the methodology used here, the improvement in properties that was shown for three-component polymers can be applied wherever “terpolymers” are used nowadays. Typically, each component contributes with a specific property; as an example, for the acrylonitrile/butadiene/styrene (ABS) plastic, the components respectively impart chemical resistance/impact resistance/rigidity, and it is used in electronic housings, auto parts, computer keyboards, etc. In fact, with this methodology, the variations required in properties (e.g., higher chemical resistance) may easily be attained by variations in the feeding profiles sustaining component interactions with the common monomer. In brief, specific properties can be obtained with improved performance.

## 4. Conclusions

A novel synthesis method was developed to prepare TCPs using free-radical emulsion polymerization. With such a method, small domain size formation at the nanophase level can be obtained. The smaller nanophase morphology of TCP versus TS allowed closer interaction of the chain components that led to a better modulus–ultimate deformation relationship that, in turn, was reflected in much higher toughness values. The difference in synthesis methodology compared with an industrial method also allowed significant changes in glass transition temperature values and mechanical performance of the polymeric materials.

With the synthesis method presented here, using dynamic and static tests for TCPs, it was established that rigidity can be sustained while toughness significantly increases, denoting synergy, and the energy dissipation capacity can be improved within a wide range of temperatures (depending on the $T_{g}$ of the homopolymers).

In summary, using such a synthesis methodology to prepare three-component polymer systems allows enhanced performance in mechanical properties, compared to the two-stage method which is typically used in industry. This methodology could be used with different monomer systems or RDRP reactions, taking into consideration the relative reactivities of the comonomers to be used for FRP (which for some systems may lead to polymer segregation) and can be easily scaled up to the industrial level. In this way, this methodology can be used to design materials for energy or sound absorption as well as plastics with high deformation capacity.

## Figures and Tables

**Table 1:** Mass content of the ingredients used for the synthesis of polymer seeds, TS polymer, and forced composition TCPs.

**Polymer Seeds (PS or PBA)**		
Ingredient	Load added (g)	Load added (mmol)
Styrene (S) or n-butyl acrylate (BA)	500	4800 or 3901
Sodium dodecyl sulfate (SDS)	10.0	35
Potassium persulfate (KPS)	10.0	37
Sodium bicarbonate (SB)	10.0	119
Distilled water	2000	-
**TS Polymeric Material**		
Ingredient	Load added (g)	Load added (mmol)
PBA latex ^a^	625	-
S	300	2881
4-vinylbenzylchloride (VBC)	75	491
SDS	7.5	26
KPS	7.5	27
SB	7.5	89
Distilled water	1500	-
**Forced Composition TCP**		
Ingredient	Load added (g)	Load added (mmol)
TCP with PS Seed		
PS latex ^a^	250	-
S	250	2400
BA	125	975
4-vinylbenzylchloride (VBC)	75	491
SDS	9.0	31
KPS	9.0	33
SB	9.0	107
Distilled water	1800	-
TCP with PBA Seed		
PBA latex ^a^	250	-
S	300	2881
BA	75	59
4-vinylbenzylchloride (VBC)	75	49
SDS	9.0	31
KPS	9.0	33
SB	9.0	107
Distilled water	1800	-

^a^ The solid polymer content was 20 wt %.

**Table 2:** Glass transition temperatures by differential scanning calorimetry and mechanodynamic analysis of TS polymer and TCP. For materials code see Section 2.2.2.

	Differential Scanning Calorimetry ^a^	Mechanodynamic Analysis ^b^	
Material	Glass Transition Temperature (°C)	First Glass Transition (°C)	Second Glass Transition (°C)
TS	108	−44	106
IPS	56	−51	58
IIPS	52	−56	54
IPBA	81	−45	77
IIPBA	76	−40	74

^a^ The measured values of $T_{g}$ were obtained with a heating ramp of 20 °C/min in nitrogen atmosphere from 0 to 120 °C; ^b^ The glass transition temperatures were obtained from the peak values in E″  plots. The frequency of mechanodynamic tests was 1 Hz and a heating rate of 1.5 °C/min.

**Table 3:** Energy dissipation capacity (A), and toughness for the TS polymer and TCPs obtained, respectively, from mechanodynamic and tensile tests. For materials code see Section 2.2.2.

Materials	Energy Dissipation Capacity ^a^ $\left(A\times 10^{-3}\right)$ (MPa °C)	Toughness ^b^ (MPa)		
		25 °C	40 °C	50 °C
TS	7.8	69 ± 20	1.1 $\times 10^{2}\pm$ 24	72 ± 17
IPS	18.8	64 ± 10	2.5 $\times 10^{3}\pm$ 2.0 $\times 10^{2}$	3.4 $\times 10^{3}\pm$ 3.4 $\times 10^{2}$
IIPS	16.2	1.0 $\times 10^{3}\pm$1.4 $\times 10^{2}$	7.5 $\times 10^{2}\pm$ 89	1.7 $\times 10^{3}\pm$ 3.2$\times 10^{2}$
IPBA	9.5	99 ± 12	1.3 $\times 10^{2}\pm$ 10	1.2 $\times 10^{2}\pm$ 31
IIPBA	10.9	42 ± 7	48 ± 26	62 ± 20

^a^ For DMA tests, the frequency was 1 Hz, and the heating rate was 1.5 °C/min. The values were estimated using the area under the loss modulus for the −60 to 50 °C interval (Figure 5b). ^b^ The crosshead speed in the tensile tests was 5 mm/min (Figure 6a–c).
